# Biomanufacturing of protective antibodies and other therapeutics in edible plant tissues for oral applications

**DOI:** 10.1111/pbi.12541

**Published:** 2016-02-13

**Authors:** Paloma Juarez, Vikram Virdi, Ann Depicker, Diego Orzaez

**Affiliations:** ^1^ Department of Plant Systems Biology VIB Gent Belgium; ^2^ Department of Plant Biotechnology and Bioinformatics Ghent University Gent Belgium; ^3^ Instituto de Biología Molecular y Celular de Plantas Consejo Superior de Investigaciones Científicas Universidad Politécnica de Valencia Valencia Spain

**Keywords:** molecular farming, edible, antibody, therapeutic protein, oral passive immunization, mucosae

## Abstract

Although plant expression systems used for production of therapeutic proteins have the advantage of being scalable at a low price, the downstream processing necessary to obtain pure therapeutic molecules is as expensive as for the traditional Chinese hamster ovary (CHO) platforms. However, when edible plant tissues (EPTs) are used, there is no need for exhaustive purification, because they can be delivered orally as partially purified formulations that are safe for consumption. This economic benefit is especially interesting when high doses of recombinant proteins are required throughout the treatment/prophylaxis period, as is the case for antibodies used for oral passive immunization (OPI). The secretory IgA (SIgA) antibodies, which are highly abundant in the digestive tract and mucosal secretions, and thus the first choice for OPI, have only been successfully produced in plant expression systems. Here, we cover most of the up‐to‐date examples of EPT‐produced pharmaceuticals, including two examples of SIgA aimed at oral delivery. We describe the benefits and drawbacks of delivering partially purified formulations and discuss a number of practical considerations and criteria to take into account when using plant expression systems, such as subcellular targeting, protein degradation, glycosylation patterns and downstream strategies, all crucial for improved yield, high quality and low cost of the final product.

## Introduction

Ever since the production of the first therapeutic protein in plants more than two decades ago, plant production platforms have matured and yielded the first commercially available plant‐made pharmaceuticals for both human and veterinary applications (Grabowski *et al*., 2014; Sack *et al*., 2015a; Yoshiola *et al*., 2012). Within the wide repertoire of therapeutic proteins, antibodies are among the most popular, due to their enormous potential for the treatment of a wide range of diseases such as certain cancers, autoimmune diseases and infectious diseases. The number and types of antibodies expressed in plants have increased incessantly since the first reports in 1989 (Hiatt *et al*., 1989), and in recent years, some plant‐made antibodies (PMAbs) have been presented as promising therapeutic solutions. For instance, the safety and immunogenicity of personalized antibodies to treat patients with non‐Hodgkin's lymphoma, produced by Large Scale Biology Corporation (McCormick *et al*., 2008) and Icon Genetics (http://www.icongenetics.com), have been demonstrated in phase I clinical trials. CaroRX^®^ (Planet Biotechnology INC, Hayward, CA, USA), an oral topical solution based on antibodies against *Streptococcus mutans* to prevent dental caries (De Muynck *et al*., 2010; Larrick *et al*., 2001; Ma, 1988; Wycoff, 2005), was evaluated in phase I and II clinical trials in the United States and has been registered as a medical device in Europe (Larrick *et al*., 1998). The first good manufacturing practice (GMP)‐compliant, plant‐derived monoclonal antibody (mAb) to undergo clinical testing in Europe was the human P2G12 against HIV‐1, which has been shown to be safe and well tolerated in healthy women when administered intravaginally (Ma *et al*., 2015). Finally, ZMapp, a cocktail comprising three individual mAbs directed against Ebola, was able to reverse Ebola disease in 100% of the infected Rhesus macaques (Qiu *et al*., 2014). The recent outbreak of Ebola in West Africa brought attention to this antibody cocktail, which was delivered to a handful of patients with their signed consent. In February 2015, ZMapp received approval from the FDA as an investigational new drug, allowing the start of clinical trials in Liberia.

Although mammalian cells (Wurm, 2004) or baculovirus‐infected insect cells (Berger *et al*., 2004) are currently the most used antibody production systems for the majority of applications, the use of transgenic plants for the expression of recombinant antibodies is gaining momentum. Accordingly, several groups compared the potential of PMAbs as antimicrobial agents with their commercial ‘biosimilars’. Zeitlin *et al*. (1998) compared a humanized antiherpes simplex virus 2 (HSV‐2) mAb expressed in mammalian cell cultures with its counterpart expressed in soybean, proving not only the similarity in their stability in mucosal secretions of the human reproductive tract, but also in the efficacy for the prevention of vaginal HSV‐2 infection in mouse. Ko *et al*. (2003) produced an anti‐rabies virus immunoglobulin G (IgG) in tobacco plants and demonstrated its effectiveness *in vivo*, showing a virus‐neutralizing activity comparable with that of its commercial counterpart. The neutralization capacity of the PMAb‐based vaginal microbicide against HIV transmission also proved to be equal or even superior to that of its counterpart produced in CHO cells (Ramessar *et al*., 2008). Moreover, competitive yields have been reported not only at a laboratory scale (Giritch *et al*., 2006; Petruccelli *et al*., 2006), but also in prototype industrial set‐ups (Bendandi *et al*., 2010; Vézina *et al*., 2009). Upscaling of PMAbs can in theory be achieved more easily and economically than with the existing animal and insect cell systems, where it requires expensive investments. Although no PMAbs are in commercial production yet and costs are difficult to estimate, it has been calculated by Planet Biotechnology that the costs of an IgA produced in plants are only 5% compared with those in mammalian cells (Daniell *et al*., 2001; Frenzel *et al*., 2013). However, the optimization of purification protocols and strict regulations on growth of transgenic plants in the field (Directive 2001/18/EC) hamper faster development.

Traits like low‐cost upscaling and ease of storage are especially promising when bulk amounts of antibodies are required, as is the case, for example for passive immunization (PI), where repeated high doses have to be applied. The protective effect of PI is appropriate for rapid responses to emergencies, such as epidemic outbreaks, protection of newborns against vertical transmission of viruses from the mother, protection of immunocompromised patients and biological warfare threats. Customized antibodies can be applied parenterally, intravenously or even better directly to the mucosae, because most infections are caused by pathogens that have a mucosal portal of entry. Antibody application via the oral route to the gastrointestinal mucosa is called oral passive immunization (OPI).

## Edible plant tissues as a platform for production of mucosal therapeutics

OPI is one of the most promising applications of PMAbs, particularly when considering that plant species with tissues that are generally recognized as safe (GRAS) may have lower purification requirements. Edible plant tissues (EPTs) could even be consumed safely, making them ideal platforms for the production of gastrointestinal therapeutics aimed at oral delivery. Although there are still very few examples of antibodies produced in EPTs, some edible mucosal therapeutics have been investigated and set a precedent.

The concept of edible plant‐made pharmaceuticals was first conceived in the early 1990s, when Charles J. Arntzen championed the idea of making vaccines in edible fruits. While visiting Bangkok, he saw a mother soothe a crying baby by offering pieces of banana, inspiring him with the idea to produce vaccines in genetically engineered food. The advantages would be enormous: yearly and local growth of plants would not only significantly reduce costs and avoid the need for refrigerated transportation over long distances, but vaccines in EPTs would also not require medical personnel or the use of syringes, which, apart from their additional cost, can be contaminated and lead to infections (Mason *et al*., 1996). Unfortunately, several limitations to the concept appeared; among others, the possible development of immunotolerance to target peptides or proteins, which has to be addressed on a case‐by‐case basis, the difficulty to control dosage requirements and the fluctuating dosage consistency, varying from fruit to fruit, plant to plant and generation to generation. To deal with the two last problems, the idea of directly consuming the fruit was abandoned and substituted by the use of dried or partially purified formulations, for which the dosage could be standardized, while sustaining low production costs (Tokuhara *et al*., 2013). Particularly, controlled doses can be achieved using lyophilized plant cells as formulation. As an example, the plant biotech company Protalix Biotherapeutics produces lyphilized carrot cells expressing either anti‐TNF antibodies or Glucocerebrosidase aimed for oral delivery (http://www.protalix.com/development-pipeline/overview-development-pipeline.asp). Other noteworthy examples of lyophilized plant cells expressing biotherapeutics for oral applications are reviewed in Chan and Daniell (2015).

Since then, a number of vaccines, antibodies and other mucosal pharmaceuticals have been produced in EPTs for oral treatments (Table 1). One of the first was a vaccine candidate based on the heat‐labile enterotoxin B (LTB) subunit of enterotoxigenic *Escherichia coli* (ETEC) produced in transgenic potato, which protected against an ETEC challenge in animal studies and was also immunogenic in humans during a phase I clinical trial (Haq *et al*., 1995; Mason *et al*., 1998; Tacket *et al*., 1998); the same was shown when it was produced in transgenic corn (Tacket, 2007; Tacket *et al*., 2004). Many examples for Alzheimer's disease, allergic diseases, or autoimmune and infectious diseases show that rice is one of the preferred EPTs for therapeutic protein production (Azegami *et al*., 2015; Takaiwa *et al*., 2015). A first example is the MucoRice‐CTB, an oral vaccine against cholera, which consists of the antigen of the cholera toxin B (CTB) accumulating in rice seed storage organelles. This vaccine was shown to induce both mucosal and systemic immunity in primates (Nochi *et al*., 2009), and also exhibited cross‐reactivity with the LTB of ETEC in piglets challenged with ETEC, when the vaccine was orally applied to the nursing sows (Takeyama *et al*., 2015). Another rice example for mucosal applications is the MucoRice‐ARP1, containing the variable domain of a rotavirus‐specific llama heavy‐chain antibody fragment, which provided efficient protection against rotavirus disease (Tokuhara *et al*., 2013). Also, the group of Takeshi Matsumura in Japan focused their interest on expressing canine interferon α in strawberries. In animal trials, they showed that canine interferon‐α that was delivered to beagles by adding dried transgenic strawberries to the dog feed had a protective and therapeutic effect against the canine periodontal disease, even at a very low dose (Yoshiola *et al*., 2012). Currently, research has advanced to the clinical trial phase (https://unit.aist.go.jp/bpri/bpri-pmt/member_e.html).

**Table 1:** Most relevant antibodies and therapeutic proteins produced in EPTs

Product	Target pathogen/disease	Plant species/EPT	Clinical status	Expression level	Reference
Capsid protein	Norwalk virus	Potato	Unknown	10–20 μg g^−1^ TW 0.23% TSP	Mason *et al*. (1996)
Heat‐labile enterotoxin B (LTB)	*E. coli*	Potato, corn	Phase I	NF	Haq *et al*. (1995)
				12.88 μg g^−1^ TW 1.85 μg mg^−1^ μg g^−1^ TP	Mason *et al*. (1998)
				3.7–15.7 μg g^−1^	Tacket *et al*. (1998)
				0.15 mg g^−1^	Tacket *et al*. (2004)
				12.88 μg g^−1^ TW 1.85 μg mg^−1^ μg g^−1^ TP	Tacket (2007)
Virus‐like particle (VLP)	Hepatitis B, Norwalk virus	Potato	Unknown	542 ng mg^−1^ TSP	Huang *et al*. (2005)
MucoRice‐CTB (Cholera toxin B subunit)	Cholera	Rice	Animal trials	30 μg seed^−1^	Nochi *et al*. (2007) Takeyama *et al*. (2015)
MucoRice‐ARP1 (Antibody)	Rotavirus	Rice	Unknown	170 μg seed^−1^	Tokuhara *et al*. (2013)
Canine interferon	Gingivitis	Strawberries	Unknown	NF	https://unit.aist.go.jp/bpri/bpri-pmt/member_e.html
VEN100 human lactoferrin	Antibiotic‐associated diarrhoea	Rice	Phase II	>5 mg g^−1^	Bethell and Huang (2004) Broz *et al*. (2013)
PRX‐112 glucocerebrosidase (GCD)	Gaucher disease	Carrot cells	Phase II	3 mg kg^−1^	www.protalix.com Shaaltiel *et al*. (2015)
PRX‐106 antibody	Tumour necrosis factor	Carrot cells	Phase I	NF	www.protalix.com
Designer antibodies	Enterotoxigenic *E. coli*	*A. thaliana* seeds	Animal trials	0.2% TSW	Virdi *et al*. (2013)
Antibody	Rotavirus	Tomato	N/A	41 μg g^−1^ FW 3.6% TSP	Juarez *et al*. (2012)

TW is tuber weight, NF is not found, TSP is total soluble protein, TP is total protein, TSW is total seed weight, FW is fresh weight and N/A is not applicable.

Several therapeutic proteins expressed in the chloroplasts of lettuce leaves also illustrate the efficiency and low cost of the production of pharmaceuticals in EPTs, even at industrial set‐ups (Kwon and Daniell, 2015). Su *et al*. (2015a) produced acid‐α glucosidase (AAG) in lettuce leaves and showed that their natural encapsulation in the chloroplast was able to avoid the adverse immune reaction occurring during the protein replacement therapy for Pompe disease when assayed in mice. Following the same rationale, Su *et al*. (2015b) also expressed the coagulation factor IX (FIX), showing the absence of pathogenic antibodies after haemophilia treatment in mice. Moreover, Shil *et al*. (2014) showed that feeding of mice with bioencapsulated ACE2/Ang‐(1–7) significantly reduced endotoxin‐induced uveitis (EIU).

As mentioned above, Protalix Biotherapeutics is engaged in the development of two new drugs naturally encapsulated within carrot cells, intended for oral delivery. One of them is the glucocerebrosidase (GCD) enzyme PRX‐112 for the treatment of Gaucher disease. Feeding experiments with rat and pig as model animals have shown that active recombinant GCD reached both liver and spleen, the target organs in the Gaucher disease (Shaaltiel *et al*., 2015). Moreover, the recombinant GCD was found to be safe and well tolerated in all 12 patients in a phase I clinical trial in Israel. The phase II clinical trial is ongoing for this product. The other carrot cell‐based protein is the anti‐tumour necrosis factor PRX‐106 for orally administered, anti‐inflammatory treatment. In preclinical studies, PRX‐106 alleviated immune‐mediated hepatitis and reduced interferon‐γ levels in a mouse model; in a phase I clinical trial, PRX‐106 has been shown to be safe and well tolerated, to have biological activity in the gut and to induce regulatory T cells.

There are some examples of antibodies produced in EPTs intended for PI at the gastrointestinal tract, two of them developed in our groups. The first one, intended for veterinary use, is the designer IgAs expressed in *Arabidopsis thaliana* seeds, consisting of a fusion between the anti‐ETEC llama heavy‐chain‐only antibodies and the Fc part of the porcine IgA. These antibodies have shown to efficiently protect ETEC‐challenged piglets against postweaning diarrhoea when administered in the form of crushed seeds mixed with the feed (Virdi *et al*., 2013). The second example consists of IgA antibodies against rotavirus produced in tomato fruits. In this work, minimally processed fruit‐derived products (tomato‐clarified extract and powder) suitable for oral intake showed anti‐VP8* binding activity and strongly inhibited virus infection in *in vitro* virus neutralization assays (Juarez *et al*., 2012).

## Secretory IgA as a target molecule for oral passive immunotherapy

Although IgA constitutes only 10–15% of the total immunoglobulin in the blood serum, it is the predominant immunoglobulin class in external secretions such as breast milk, saliva, tears and the mucosal surfaces of the bronchial, genitourinary and digestive tracts. The daily human production of 5–15 g of SIgA into mucous secretions is greater than that of any other immunoglobulin class (Goldsby *et al*., 2003). SIgA serves as the first line of defence at mucosal surfaces by neutralizing pathogenic microorganisms. Because of its polymeric form, SIgA can cross‐link large antigens with multiple epitopes. Binding of SIgA to bacterial and viral surface antigens prevents attachment of the pathogens to mucosal cells, thus inhibiting viral infection and bacterial colonization. Complexes of SIgAs and antigens are easily entrapped in the mucus and then eliminated by the ciliated epithelial cells of the respiratory and genitourinary tract or by peristalsis of the gut. For this reason, SIgA is the antibody isotope best suited for OPI (Corthésy, 2003, 2010, 2013).

In spite of all obvious advantages that SIgA presents as a therapeutic molecule, its recombinant production has rarely been attempted for commercial purposes, probably due to the technical difficulties associated with the expression, assembly and recovery of a protein complex composed of four different polypeptides [heavy chain (HC), light chain (LC), joining chain and secretory component (SC)] and comprising a total of 10 individual monomers. Under physiological conditions, the biosynthesis of a SIgA complex requires the cooperation between plasma cells, which produce dimeric IgAs (dIgAs), and mucosal epithelial cells that bind the dIgAs via the polymeric immunoglobulin receptor (pIgR). During transcytosis, the pIgR is cleaved, resulting in the secretion of the SIgA with the IgA‐bound pIgR piece, called SC, wrapped around the IgA. Although some attempts to produce a correctly assembled SIgA by co‐expressing all four different genes in a single mammalian cell type have been successful (Chintalacharuvu and Morrison, 1997), the yields need to be improved and the technology is yet to be developed to achieve a commercially viable product (Renukuntla *et al*., 2013).

The first attempt to produce a plant‐made SIgA for PI was the murine hybrid Guy's 13 (Ma *et al*., 1995). This technology was later acquired by Planet Biotechnology (Larrick *et al*., 2001), evaluated in phase I and II clinical trials and registered as the medical device called CaroRX^®^ (Weintraub *et al*., 2005). Since then, just a few research groups have reported the expression of SIgA in heterologous systems. Wieland *et al*. (2005) successfully expressed chicken SIgAs against *Eimeria acervulina* in *Nicotiana benthaminana* leaves. More recently, Virdi *et al*. (2013) produced SIgA‐like antibodies against ETEC in *A. thaliana* seeds. Recently, we also demonstrated the relevance of the proper optimization of SIgA production in a case‐by‐case basis, by performing a combinatorial assembly and analysis of 16 versions of SIgA against rotavirus transiently expressed in *N. benthamiana* (Juarez *et al*., 2013; Sarrion‐Perdigones *et al*., 2011a). Paul *et al*. (2014) expressed and characterized a human SIgA format of the anti‐HIV monoclonal antibody 2G12 using both transgenic tobacco plants and transient expression in *N. benthamiana* as expression hosts.

Because the production of human SIgA antibodies at a commercially viable scale in animal cells remains an unsolved technological problem, plant‐based platforms, and especially those using EPTs when SIgA production is intended for OPI, appear to be the most promising candidates.

## Practical considerations

As for every therapeutic which is envisioned to be produced in plants, the design of a plant biofactory for antibody production requires multiple considerations involving not only the expression levels of the antibody, but also additional aspects, such as bio‐availability, transformation strategy (Obembe *et al*., 2011), subcellular targeting, protein degradation, glycosylation patterns and downstream strategies, all of them influencing the yield, quality and cost of the final product (Sarrion‐Perdigones *et al*., 2011b). To date, most of these aspects have been addressed separately, mainly on an empirical basis. Thus, future optimizations will probably require designs that integrate all of them following a global approach.

One of the main challenges of oral delivery of molecules intended to reach the blood stream is their efficient absorption. Upon oral administration, the gut epithelium acts as a physical and biochemical barrier for absorption of proteins, resulting in low bioavailability. Although this remains an unsolved problem, certain approaches, such as chemical modification, use of absorption and penetration enhancers, use of muco‐adhesive polymers and covalent conjugation to carrier molecules such as the (nontoxic) CTB enabling recognition of a target receptor, can enhance the oral bioavailability of peptides. However, these approaches, together with the understanding of the tertiary structures of macromolecules, need to be evaluated on a case‐by‐case basis to efficiently improve the bioavailability of each target protein (Kwon and Daniell, 2015; Renukuntla *et al*., 2013).

Another important challenge is achieving high expression levels, however, until now a feasible solution resolving this requirement has not been found. Although the transient expression system in *Nicotiana benthamiana* is the fastest strategy for recombinant protein production, the yields achieved are not sufficient for obtaining the large quantities of antibodies required for PI strategies. Moreover, it is not the most adequate system for production of therapeutic proteins for oral intake of partially purified formulations, because tobacco leaves lack the GRAS status. On the contrary, when using the seeds of stably transformed plants as a production platform, higher yields can be obtained due to their high protein content‐nature and low water content (Table 1). If even higher amounts are required, these can be achieved by incorporating a process of lyophilization of different plant tissues. By removing the water content, the concentration of the target protein can be increased more than 20‐fold (Chan and Daniell, 2015).

Although extracellular secretion is the natural route for antibodies in mammals, targeting antibody chains to specific compartments in the plant cell can result in advantages in terms of stability, yield or downstream processing (Arcalis *et al*., 2013; De Muynck *et al*., 2010). Among the different compartments that have been tested as destination for recombinant antibodies (chloroplast, protein storage vacuole, etc.), the secretory pathway seems to be the most convenient route for correct antibody folding and assembly, due to the oxidizing environment required for formation of disulphide bonds, the low abundance of proteases and the presence of molecular chaperones found in the ER (Ma *et al*., 2003). Antibody chains are targeted to the plant secretory pathway using an appropriate N‐terminal signal peptide, which is either a plant signal peptide (De Buck *et al*., 2012), or the native signal peptide of the Ig (Hiatt *et al*., 1989; Sainsbury *et al*., 2008), which works as efficiently in many cases (De Buck *et al*., 2012; Hiatt *et al*., 1989; Sainsbury *et al*., 2008). Once in the cis‐Golgi, antibodies can either be efficiently retrieved back to the ER using a C‐terminal H/KDEL retention signal, or secreted into the apoplast, downstream of the secretory pathway (De Muynck *et al*., 2009; Petruccelli *et al*., 2006). Although several antibodies have been reported to accumulate well in the apoplast (De Muynck *et al*., 2009; De Wilde *et al*., 1998; Düring *et al*., 1990), retention in the ER is often preferred for yield improvement and for dodging plant‐specific, Golgi‐derived N‐glycosylation with β‐1,2‐xylose and core α‐1,3‐fucose that could cause immunogenicity in target organisms (Bencúrová *et al*., 2004; Gomord *et al*., 2010). However, as reported by De Meyer *et al*. (2015) and De Meyer and Depicker (2014), the final protein destination of H/KDEL‐tagged recombinant proteins is unpredictable and, hence, plant‐specific N‐glycans cannot be effectively avoided following this strategy. On another front, the progress in plant glycoengineering has made possible to produce antibodies with customized and highly homogenous glycosylation patterns, which can lead to higher product quality and clinical efficacy (Bosch *et al*., 2013; Castilho and Steinkellner, 2012; Castilho *et al*., 2012; Steinkellner and Castilho, 2015). Different strategies have been followed to express recombinant proteins with a mammalian‐like N‐glycosylation pattern, such as the down‐regulation of endogenous α‐1,3‐fucosyltransferase (FT) and β‐1,2‐xylosyltransferase (XT), the expression of a chimeric form of the human β1,4‐galactosyltransferase (GalT) targeted to a late Golgi compartment (Bakker *et al*., 2001; Strasser *et al*., 2009) and the addition of terminal sialic residues, by engineering of the full mammalian *N‐*acetylneuraminic acid (Neu5Ac) biosynthesis pathway (Castilho *et al*., 2008, 2010; Loos and Steinkellner, 2014). When glycan adaptation strategies were used for production of antibodies, the resulting glycoforms were highly homogeneous and showed a dramatic increase in the antibody‐dependent cellular cytotoxicity (ADCC) activity (Whaley *et al*., 2011) which points out the benefits of plant‐to‐human modification of the glycan structure.

Recently, a novel and different approach emerged to efficiently get rid of plant‐specific glycosylation. GlycoDelete, a strategy previously developed for its use in mammalian cells (Meuris *et al*., 2014), consists on expressing a plant Golgi‐targeted version of the endo‐*N*‐acetyl‐β‐D‐glucosaminidase (endoT) from the fungus *Hypocrea jecorina* in mutant plants lacking *N*‐acetylglucosaminyltransferase‐I (GnTI) activity. These mutants only accumulate high‐mannose glycans which are substrates for the endoT enzyme. Consequently, the endoT releases the glycan structure from the target protein, leaving the innermost GlcNAc (which is often important to maintain the overall protein fold) attached to the amino acid backbone (Piron *et al*., 2015).

Even if it is advisable to mimic mammal glycosylation in injected therapeutic proteins to avoid unwanted antigenicity, when the therapeutic proteins are aimed at oral treatment, antigenicity against the sugars is probably not so determining. Because we daily consume plant‐specific glycans in our diet, plant glycans attached to these recombinant proteins are not expected to generate unexpected allergic reactions. Moreover, the concern of regulatory agencies about glycosylation patterns has probably decreased significantly after the commercialization of ELELYSO^®^ (Protalix Biotherapeutics, Carmiel, Israel), the injectable GCD from Protalix Biotherapeutics, produced in carrot cell suspensions, which showed good results in clinical trials in spite of carrying plant‐specific xylose and fucose residues (Aviezer *et al*., 2009; Cox, 2010; Shaaltiel *et al*., 2007).

As stated above, specific glycosylation patterns can also improve the stability of antibodies. Plants are heterologous environments for antibodies, and proteases may affect the integrity of these during both protein accumulation and protein extraction. Antibodies may undergo complete hydrolysis, directly reducing the final yield, or partial degradation, which can alter the integrity and activity of the final product (Benchabane *et al*., 2008; Faye *et al*., 2005; Hehle *et al*., 2015). Together with obtaining a proper glycosylation pattern of the target antibody, other strategies have been reported to increase both the yield and quality (nondegraded) of PMAbs. For instance, it has been proposed that a balanced co‐expression of heavy and light chains is another clue factor for achieving high yields, because unassembled antibody chains, which are retained by the ER‐resident chaperone BiP, could be degraded via plant ER‐associated degradation systems (Liu and Li, 2014). For this, stronger promoters should be used to boost the expression of antibody chains that are limiting the generation of complete antibody complexes. Other approaches are the use of tissue‐specific promoters to confine transgene expression to compartments with reduced metabolic activity or targeting proteins to specific cellular organelles. Gene knockout or silencing of plant peptidases is also a tool to take into account if there is a single or only a few target peptidases, which are not essential for plant growth (De Muynck *et al*., 2010). For the latter, the specific proteases that are active in the targeted time/space must be detected. Activity‐based probes are being developed, which react with the active sites of different enzymes – proteases in this case – in an activity‐dependent manner. Proteases can then be identified and further knocked out if required (Ilyas *et al*., 2015; Lu *et al*., 2015). Finally, the co‐expression of recombinant protease inhibitors interfering with endogenous proteases has also been proposed (Benchabane *et al*., 2008; Robert *et al*., 2013). Apart from the *in planta* stability, there is also a high concern regarding antibody stability in the harsh conditions of the gastrointestinal tract when these antibodies are aimed for oral delivery. In this framework, the glycosylation of the antibodies, and more concretely in the hinge region, also plays a key role (Kim *et al*., 1994; Plomp *et al*., 2015). Another advantage of PMAbs that falls within this context is that in partially purified formulations for oral delivery, antibodies are protected by the plant tissue matrix. The plant cell wall provides a natural barrier to the harsh conditions of the gastrointestinal gut that can be further improved by targeting the antibodies to different cell compartments (Sack *et al*., 2015a). This benefit is also applicable for proteins produced in plant cell suspensions, as it is the case for the above‐mentioned therapeutic proteins from Protalix Biotherapeutics, which are naturally encapsulated within the carrot cells.

Of course, the use of minimally processed formulations relies on the logical expectation that the edible status of the transgenic plant tissue remains unaltered. However, it has been repeatedly argued that transgenesis leads to unintended effects in the final composition of the plant tissues, such as changes based on the integration of the transgene, biological interactions caused by transgene‐encoded proteins or spurious somaclonal mutations (Araki and Ishii, 2015; Heinemann *et al*., 2011; Schnell *et al*., 2015). Although unintended effects have become one of the most controversial questions about biological safety of genetically modified organisms (GMOs), risk analysis studies and detailed GMO characterizations performed until now have not revealed any harmful unintended effect (García‐Cañas *et al*., 2011; Ladics *et al*., 2015). There are several recent publications that assess the edibility of genetically engineered EPTs producing proteins aimed for pharmaceutical use, one of them being of our group (Juarez *et al*., 2014). In this work, as a way to evaluate the changes in the tomato composition introduced by IgA production, the proteomic and metabolomic profiles of transgenic tomatoes expressing human IgA were analysed and compared with those of nontransgenic fruits. Although this work itself cannot rule out the possibility of harmful unintended effects associated with every single strategy involving IgA production in fruits, the data suggest that the formulations derived from these IgA tomatoes are as safe for consumption as equivalent formulations derived from wild‐type tomatoes. A second work, performed by Kurokawa *et al*. (2013), describes that no known rice allergens appear to be up‐regulated by genetic modification of MucoRice‐CTB, suggesting that MucoRice‐CTB has the potential as a safe oral cholera vaccine. These two studies are the first step for confirming the safety of transgenic plants – or their edible tissues – producing recombinant proteins for mucosal applications. When analysing the unintended effects produced in GMOs, it is also useful to take into account a whole‐genome and transcriptomic analysis. In this framework, Kawakatsu *et al*. (2013) conducted an analysis where the genome of a rice line expressing a seed‐based edible vaccine containing two pollen allergens was compared with the genome of its host line. In this study, the authors found that the differences between both genomes were minor in comparison with those found when comparing the parental line with other related rice cultivars, pointing out that most of the alterations corresponded to somaclonal variation. Moreover, when performing strand‐specific mRNA‐Seq, similar transcriptomes were revealed for both transgenic and parental lines, supporting genomic integrity between them.

Another important consideration is how to handle minimally processed plant preparations or partially purified drug substances to control the quality (e.g. batch‐to‐batch consistency) of the recombinant pharmaceutical products according to GMP compliance. The procedures for the GMP/GACP‐compliant upstream production of 2G12 in transgenic tobacco plants have been recently summarized by Sack *et al*. (2015b). In general, the manufacturing of recombinant biologically active substances in plants is covered at the EU level by the guideline EMEA/CHMP/BWP/48316/2006 (http://www.ema.europa.eu/docs/en_GB/document_library/Scientific_guideline/2009/09/WC500003154.pdf), which basically adapts aspects of the quality guidance already in place for other production systems to the special case of transgenic plants. Although this guideline covers several technical aspects, such as generation of transformants, banking systems, upstream and downstream processing, characterization of active ingredients and control of contamination with adventitious agents, it does not specifically address minimally or partially processed products. It states, however, that process‐related impurities (e.g. host cell proteins, host cell DNA, reagents, downstream impurities) are expected to differ qualitatively among different manufacturing processes, and therefore, the qualitative comparison of these parameters may not be relevant in bio‐similar comparability exercises.

In the absence of specific guidelines covering recombinant biologics in minimally processed plant tissues, it can be useful to review and compare the existing guidelines for related products with similar characteristics, such as herbal medicinal preparations containing identified active ingredients (e.g. powdered herbal substances, extracts, essential oils, expressed juices and processed exudates among others). For those products, active ingredients are defined as those chemically defined substances or groups of substances which are generally accepted to contribute substantially to the therapeutic activity, a description that could be easily assimilated to recombinant biologics made in plants. To ensure appropriate and consistent quality of medicinal plant/herbal substances, the Guideline EMEA/HMPC/246816/2005 (http://www.ema.europa.eu/docs/en_GB/document_library/Scientific_guideline/2011/09/WC500113210.pdf) establishes good agricultural and collection practices (GACP) for herbal starting materials. Furthermore, the GMP concept for the manufacture, processing, packaging and storage of active pharmaceutical ingredients also applies to medicinal plants/herbal substances as covered by EU‐GMP Guideline Part II (http://www.ecv.de/leseproben/781807.pdf). Moreover, in the case of herbal preparations with constituents of known therapeutic activity, and besides GACP and GMP considerations, quality assays for marketing authorization include tests of microbial limits, pesticides and micotoxins, and specifically require identification of functional components with validated, specific, stability‐indicating assays, along with details of the used analytical procedure(s) (http://www.ema.europa.eu/docs/en_GB/document_library/Scientific_guideline/2011/09/WC500113209.pdf). Eventually, similar specific assays (e.g. ELISA tests, virus neutralization assays) should be adapted and implemented for recombinant products to ensure batch‐to‐batch consistency and stability of active ingredients, that is recombinant antibodies or biologics. Finally, as an additional consideration regarding authorization strategies, it should be noted that herbal preparations with active ingredients for topical application (e.g. semi‐solid formulations) are frequently certified as medical devices and not as topical drugs. Medical devices are those that primarily unfold their therapeutic effect by physical means and do not achieve their principal intended action in or on the human body by pharmacological, immunological or metabolic means, but which may be assisted by such means. As mentioned above, CaroRX^®^ received marketing approval in the EU as a medical device, and similar authorization strategies could be probably explored for other semi‐purified mucosal passive agents against, for example, skin or oral diseases, as they provide a less complex marketing authorization process compared with topical and oral drugs (Korting and Schollmann, 2012).

## Final remarks

Plants are emerging systems for the production of recombinant antibodies. Despite all advantages that plants can offer as antibody expression systems, so far, very few antibodies have been subjected to clinical trials (Sack *et al*., 2015a). There are two main reasons why PMAbs tend to get trapped in the interface between animal trials and commercialization. First, because mammalian systems are already established and have the dominant share of the market, they somehow obstruct the natural development of new expression systems, even if these have striking advantages. Second, the regulatory framework for the field cultivation of genetically modified plants is highly demanding, particularly in Europe, as described in the directive 2001/18/EC and its amendment 2008/27/EC.

In all probability, plant antibody expression systems are not going to replace the mammalian ones. These systems have well‐established production protocols for many antibodies, which have already gone through the current GMP production and drug regulations. Production of ‘biosimilars’ in plants will most likely have to go through strong competition to reach the market. Therefore, the market of PMAbs will probably have more opportunities with the production of ‘biobetters’ (therapeutics with enhanced features and/or efficacy) and of complex molecules that cannot be produced in mammalian platforms, such as SIgA or IgM, which can be exploited for mucosal therapies. Plant platforms present benefits as being human pathogen free, ensuring rapid availability when using transient expression systems, providing *à la carte* glycosylation patterns and having low production and processing costs. Moreover, if EPTs are used, the final cost of manufacturing can be dramatically reduced, because exhaustive purification would no longer be needed for certain applications. Edible plant tissues with GRAS status (such as fruits, seeds and certain leaves) can therefore be utilized as reservoirs of antibodies such as SIgAs for easy long‐time storage and enabling quick availability after minimal processing when required for OPI. In the case of seeds, due to their low water content, they can be stored for years without losing antibody efficacy (Azegami *et al*., 2015; Virdi *et al*., 2013). If using more watery tissues, such as fleshy fruits and leaves, a step of lyophilization or spray‐drying should be included prior to storing (Juarez *et al*., 2012; Su *et al*., 2015b). When using partially purified products derived from EPTs, it is important to take into account that an edibility and allergenicity assessment should be performed to ensure that the plant‐derived products are convenient for oral use.

Overall, plant expression systems are evolving and finding their niche to be complementary to other established expression systems. In this framework, EPTs – either seeds or dried tissues – that offer the possibility of long‐lasting, refrigeration‐free storage and low cost of production and downstream processing, will also be a convenient option for developing countries that cannot afford costly therapies and/or prophylaxis for certain infectious diseases.

## References

[pbi12541-bib-0001] Araki, M. and Ishii, T. (2015) Towards social acceptance of plant breeding by genome editing. Trends Plant Sci. 20, 145–149. 25726138 10.1016/j.tplants.2015.01.010

[pbi12541-bib-0002] Arcalis, E. , Stadlmann, J. , Rademacher, T. , Marcel, S. , Sack, M. , Altmann, F. and Stoger, E. (2013) Plant species and organ influence the structure and subcellular localization of recombinant glycoproteins. Plant Mol. Biol. 83, 105–117. 23553222 10.1007/s11103-013-0049-9

[pbi12541-bib-0003] Aviezer, D. , Brill‐Almon, E. , Shaaltiel, Y. , Hashmueli, S. , Bartfeld, D. , Mizrachi, S. , Liberman, Y. *et al*. (2009) A plant‐derived recombinant human glucocerebrosidase enzyme — a preclinical and phase I investigation. PLoS ONE, 4, e4792. 19277123 10.1371/journal.pone.0004792 PMC2652073

[pbi12541-bib-0004] Azegami, T. , Itoh, H. , Kiyono, H. and Yuki, Y. (2015) Novel transgenic rice‐based vaccines. Arch. Immunol. Ther. Exp. (Warsz.) 63, 87–99. 25027548 10.1007/s00005-014-0303-0

[pbi12541-bib-0005] Bakker, H. , Bardor, M. , Molthoff, J.W. , Gomord, V. , Elbers, I. , Stevens, L.H. , Jordi, W. *et al*. (2001) Galactose‐extended glycans of antibodies produced by transgenic plants. Proc. Natl. Acad. Sci. USA, 98, 2899–2904. 11226338 10.1073/pnas.031419998 PMC30237

[pbi12541-bib-0006] Benchabane, M. , Goulet, C. , Rivard, D. , Faye, L. , Gomord, V. and Michaud, D. (2008) Preventing unintended proteolysis in plant protein biofactories. Plant Biotechnol. J. 6, 633–648. 18452504 10.1111/j.1467-7652.2008.00344.x PMC7159130

[pbi12541-bib-0007] Bencúrová, M. , Hemmer, W. , Focke‐Tejkl, M. , Wilson, I.B.H. and Altmann, F. (2004) Specificity of IgG and IgE antibodies against plant and insect glycoprotein glycans determined with artificial glycoforms of human transferrin. Glycobiology, 14, 457–466. 15033940 10.1093/glycob/cwh058

[pbi12541-bib-0008] Bendandi, M. , Marillonnet, S. , Kandzia, R. , Thieme, F. , Nickstadt, A. , Herz, S. , Fröde, R. *et al*. (2010) Rapid, high‐yield production in plants of individualized idiotype vaccines for non‐Hodgkin's lymphoma. Ann. Oncol. 21, 2420–2427. 20494963 10.1093/annonc/mdq256

[pbi12541-bib-0009] Berger, I. , Fitzgerald, D.J. and Richmond, T.J. (2004) Baculovirus expression system for heterologous multiprotein complexes. Nat. Biotechnol. 22, 1583–1587. 15568020 10.1038/nbt1036

[pbi12541-bib-0010] Bethell, D.R. and Huang, J. (2004) Recombinant human lactoferrin treatment for global health issues: iron deficiency and acute diarrhea. Biometals, 17, 337–342. 15222487 10.1023/b:biom.0000027714.56331.b8

[pbi12541-bib-0011] Bosch, D. , Castilho, A. , Loos, A. , Schots, A. and Steinkellner, H. (2013) *N*‐glycosylation of plant‐produced recombinant proteins. Curr. Pharm. Des. 19, 5503–5512. 23394562 10.2174/1381612811319310006

[pbi12541-bib-0012] Broz, A. , Huang, N. and Unruh, G. (2013) Plant‐based protein biomanufacturing. Genet. Eng. Biotechnol. News, 33, 32–33.

[pbi12541-bib-0013] Castilho, A. and Steinkellner, H. (2012) Glyco‐engineering in plants to produce human‐like *N*‐glycan structures. Biotechnol. J. 7, 1088–1098. 22890723 10.1002/biot.201200032

[pbi12541-bib-0014] Castilho, A. , Pabst, M. , Leonard, R. , Veit, C. , Altmann, F. , Mach, L. , Glossl, J. *et al*. (2008) Construction of a functional CMP‐sialic acid biosynthesis pathway in Arabidopsis. Plant Physiol. 147, 331–339. 18326787 10.1104/pp.108.117572 PMC2330290

[pbi12541-bib-0015] Castilho, A. , Strasser, R. , Stadlmann, J. , Grass, J. , Jez, J. , Gattinger, P. , Kunert, R. *et al*. (2010) In planta protein sialylation through overexpression of the respective mammalian pathway. J. Biol. Chem. 285, 15923–15930. 20305285 10.1074/jbc.M109.088401 PMC2871460

[pbi12541-bib-0016] Castilho, A. , Neumann, L. , Daskalova, S. , Mason, H.S. , Steinkellner, H. , Altmann, F. and Strasser, R. (2012) Engineering of sialylated mucin‐type *O*‐glycosylation in plants. J. Biol. Chem. 287, 36518–33626. 22948156 10.1074/jbc.M112.402685 PMC3476317

[pbi12541-bib-0017] Chan, H.T. and Daniell, H. (2015) Plant‐made oral vaccines against human infectious diseases‐Are we there yet? Plant Biotechnol. J. 13, 1056–1070. 26387509 10.1111/pbi.12471 PMC4769796

[pbi12541-bib-0018] Chintalacharuvu, K.R. and Morrison, S.L. (1997) Production of secretory immunoglobulin A by a single mammalian cell. Proc. Natl. Acad. Sci. USA, 94, 6364–6368. 9177223 10.1073/pnas.94.12.6364 PMC21055

[pbi12541-bib-0019] Corthésy, B. (2003) Recombinant secretory immunoglobulin A in passive immunotherapy: linking immunology and biotechnology. Curr Pharm Biotechnol. 4, 51–67. 12570682 10.2174/1389201033378020

[pbi12541-bib-0020] Corthésy, B. (2010) Role of secretory immunoglobulin A and secretory component in the protection of mucosal surfaces. Future Microbiol. 5, 817–829. 20441552 10.2217/fmb.10.39

[pbi12541-bib-0021] Corthésy, B. (2013) Role of secretory IgA in infection and maintenance of homeostasis. Autoimmun. Rev. 12, 661–665. 23201924 10.1016/j.autrev.2012.10.012

[pbi12541-bib-0022] Cox, T.M. (2010) Gaucher disease: clinical profile and therapeutic developments. Biologics, 4, 299–313. 21209725 10.2147/BTT.S7582 PMC3010821

[pbi12541-bib-0023] Daniell, H. , Streatfield, S.J. and Wycoff, K. (2001) Medical molecular farming: production of antibodies, biopharmaceuticals and edible vaccines in plants. Trends Plant Sci. 6, 219–226. 11335175 10.1016/S1360-1385(01)01922-7 PMC5496653

[pbi12541-bib-0024] De Buck, S. , Virdi, V. , De Meyer, T. , De Wilde, K. , Piron, R. , Nolf, J. , Van Lerberge, E. *et al*. (2012) Production of camel‐like antibodies in plants. Methods Mol. Biol. 911, 305–324. 22886260 10.1007/978-1-61779-968-6_19

[pbi12541-bib-0025] De Meyer, T. and Depicker, A. (2014) Trafficking of endoplasmic reticulum‐retained recombinant proteins is unpredictable in *Arabidopsis thaliana* . Front. Plant Sci. 5, 473. 25309564 10.3389/fpls.2014.00473 PMC4163989

[pbi12541-bib-0026] De Meyer, T. , Laukens, B. , Nolf, J. , Van Lerberge, E. , De Rycke, R. , De Beuckelaer, A. , De Buck, S. *et al*. (2015) Comparison of VHH‐Fc antibody production in *Arabidopsis thaliana*,* Nicotiana benthamiana* and *Pichia pastoris* . Plant Biotechnol. J. 13, 938–947. 25641071 10.1111/pbi.12330

[pbi12541-bib-0027] De Muynck, B. , Navarre, C. , Nizet, Y. , Stadlmann, J. and Boutry, M. (2009) Different subcellular localization and glycosylation for a functional antibody expressed in *Nicotiana tabacum* plants and suspension cells. Transgenic Res. 18, 467–482. 19140023 10.1007/s11248-008-9240-1

[pbi12541-bib-0028] De Muynck, B. , Navarre, C. and Boutry, M. (2010) Production of antibodies in plants: status after twenty years. Plant Biotechnol. J. 8, 529–563. 20132515 10.1111/j.1467-7652.2009.00494.x

[pbi12541-bib-0029] De Wilde, C. , De Rycke, R. , Beeckman, T. , De Neve, M. , Van Montagu, M. , Engler, G. and Depicker, A. (1998) Accumulation pattern of IgG antibodies and Fab fragments in transgenic *Arabidopsis thaliana* plants. Plant Cell Physiol. 39, 639–646. 9697345 10.1093/oxfordjournals.pcp.a029416

[pbi12541-bib-0030] Düring, K. , Hippe, S. , Kreuzaler, F. and Schell, J. (1990) Synthesis and self‐assembly of a functional monoclonal antibody in transgenic *Nicotiana tabacum* . Plant Mol. Biol. 15, 281–293. 2129424 10.1007/BF00036914

[pbi12541-bib-0031] Faye, L. , Boulaflous, A. , Benchabane, M. , Gomord, W. and Michaud, D. (2005) Protein modifications in the plant secretory pathway: current status and practical implications in molecular pharming. Vaccine. 23, 1770–1778. 15734039 10.1016/j.vaccine.2004.11.003

[pbi12541-bib-0032] Frenzel, A. , Hust, M. and Schirrmann, T. (2013) Expression of recombinant antibodies. Front Immunol. 4, 217. 23908655 10.3389/fimmu.2013.00217 PMC3725456

[pbi12541-bib-0033] García‐Cañas, V. , Simó, C. , León, C. , Ibañez, E. and Cifuentes, A. (2011) MS‐based analytical methodologies to characterize genetically modified crops. Mass Spectrom. Rev. 30, 396–416. 21500243 10.1002/mas.20286

[pbi12541-bib-0034] Giritch, A. , Marillonnet, S. , Engler, C. , van Eldik, G. , Botterman, J. , Klimyuk, V. and Gleba, Y. (2006) Rapid high‐yield expression of full‐size IgG antibodies in plants coinfected with noncompeting viral vectors. Proc. Natl. Acad. Sci. USA, 103, 14701–14706. 16973752 10.1073/pnas.0606631103 PMC1566189

[pbi12541-bib-0035] Goldsby, R.A. , Kindt, T.J. , Osborne, B.A. and Kuby, J . (2003) Immunology. New York: W.H. Freeman and Company.

[pbi12541-bib-0036] Gomord, V. , Fitchette, A.‐C. , Menu‐Bouaouiche, L. , Saint‐Jore‐Dupas, C. , Plasson, C. , Michaud, D. and Faye, L. (2010) Plant‐specific glycosylation patterns in the context of therapeutic protein production. Plant Biotechnol. J. 8, 564–587. 20233335 10.1111/j.1467-7652.2009.00497.x

[pbi12541-bib-0037] Grabowski, G.A. , Golembo, M. and Shaaltiel, Y. (2014) Taliglucerase alfa: an enzyme replacement therapy using plant cell expression technology. Mol. Genet. Metab. 112, 1–8. 24630271 10.1016/j.ymgme.2014.02.011

[pbi12541-bib-0038] Haq, T.A. , Mason, H.S. , Clements, J.D. and Arntzen, C.J. (1995) Oral immunization with a recombinant bacterial antigen produced in transgenic plants. Science, 268, 714–716. 7732379 10.1126/science.7732379

[pbi12541-bib-0039] Hehle, V.K. , Lombardi, R. , van Dolleweerd, C.J. , Paul, M.J. , Di Micco, P. , Morea, V. , Benvenuto, E. *et al*. (2015) Site‐specific proteolytic degradation of IgG monoclonal antibodies expressed in tobacco plants. Plant Biotechnol. J. 13, 235–245. 25283551 10.1111/pbi.12266

[pbi12541-bib-0040] Heinemann, J.A. , Kurenbach, B. and Quist, D. (2011) Molecular profiling — a tool for addressing emerging gaps in the comparative risk assessment of GMOs. Environ. Int. 37, 1285–1293. 21624662 10.1016/j.envint.2011.05.006

[pbi12541-bib-0041] Hiatt, A. , Cafferkey, R. and Bowdish, K. (1989) Production of antibodies in transgenic plants. Nature, 342, 76–78. 2509938 10.1038/342076a0

[pbi12541-bib-0042] Huang, Z. , Elkin, G. , Maloney, B.J. , Beuhner, N. , Arntzen, C.J. , Thanavala, Y. and Mason, H.S. (2005) Virus‐like particle expression and assembly in plants: hepatitis B and Norwalk viruses. Vaccine. 23, 1851–1858. 15734055 10.1016/j.vaccine.2004.11.017

[pbi12541-bib-0043] Ilyas, M. , Hörger, A.C. , Bozkurt, T.O. , van den Burg, H.A. , Kaschani, F. , Kaiser, M. , Belhaj, K. *et al*. (2015) Functional divergence of two secreted immune proteases of tomato. Curr. Biol. 25, 2300–2306. 26299516 10.1016/j.cub.2015.07.030

[pbi12541-bib-0044] Juarez, P. , Presa, S. , Espí, J. , Pineda, B. , Antón, M.T. , Moreno, V. , Buesa, J. *et al*. (2012) Neutralizing antibodies against rotavirus produced in transgenically labelled purple tomatoes. Plant Biotechnol. J. 10, 341–352. 22070155 10.1111/j.1467-7652.2011.00666.x

[pbi12541-bib-0045] Juarez, P. , Huet‐Trujillo, E. , Sarrion‐Perdigones, A. , Falconi, E.E. , Granell, A. and Orzaez, D. (2013) Combinatorial analysis of secretory immunoglobulin A (sIgA) expression in plants. Int. J. Mol. Sci. 14, 6205–6222. 23507755 10.3390/ijms14036205 PMC3634489

[pbi12541-bib-0046] Juarez, P. , Fernandez‐del‐Carmen, A. , Rambla, J.L. , Presa, S. , Mico, A. , Granell, A. and Orzaez, D. (2014) Evaluation of unintended effects in the composition of tomatoes expressing a human immunoglobulin A against rotavirus. J. Agric. Food Chem. 62, 8158–8168. 25065456 10.1021/jf502292g

[pbi12541-bib-0047] Kawakatsu, T. , Kawahara, Y. , Itoh, T. and Takaiwa, F. (2013) A whole‐genome analysis of a transgenic rice seed‐based edible vaccine against cedar pollen allergy. DNA Res. 20, 623–631. 23956243 10.1093/dnares/dst036 PMC3859328

[pbi12541-bib-0048] Kim, H. , Yamaguchi, Y. , Masuda, K. , Matsunaga, C. , Yamamoto, K. , Irimura, T. , Takahashi, N. *et al*. (1994) *O*‐glycosylation in hinge region of mouse immunoglobulin G2b. J. Biol. Chem. 269, 12345–12350. 7512967

[pbi12541-bib-0049] Ko, K. , Tekoah, Y. , Rudd, P.M. , Harvey, D.J. , Dwek, R.A. , Spitsin, S. , Hanlon, C.A. *et al*. (2003) Function and glycosylation of plant‐derived antiviral monoclonal antibody. Proc. Natl. Acad. Sci. USA, 100, 8013–8018. 12799460 10.1073/pnas.0832472100 PMC164704

[pbi12541-bib-0050] Korting, H.C. and Schollmann, C. (2012) Medical devices in dermatology: topical semi‐solid formulations for the treatment of skin diseases. J. Dtsch. Dermatol. Ges. 10, 103–109. 21851553 10.1111/j.1610-0387.2011.07764.x

[pbi12541-bib-0051] Kurokawa, S. , Nakamura, R. , Mejima, M. , Kozuka‐Hata, H. , Kuroda, M. , Takeyama, N. , Oyama, M. *et al*. (2013) MucoRice‐cholera toxin B‐subunit, a rice‐based oral cholera vaccine, down‐regulates the expression of α‐amylase/trypsin inhibitor‐like protein family as major rice allergens. J. Proteome Res. 12, 3372–3382. 23763241 10.1021/pr4002146

[pbi12541-bib-0052] Kwon, K.C. and Daniell, H. (2015) Low‐cost oral delivery of protein drugs bioencapsulated in plant cells. Plant Biotechnol. J. 13, 1017–1022. 26333301 10.1111/pbi.12462 PMC4769795

[pbi12541-bib-0053] Ladics, G.S. , Bartholomaeus, A. , Bregitzer, P. , Doerrer, N.G. , Gray, A. , Holzhauser, T. , Jordan, M. *et al*. (2015) Genetic basis and detection of unintended effects in genetically modified crop plants. Transgenic Res. 24, 587–603. 25716164 10.1007/s11248-015-9867-7 PMC4504983

[pbi12541-bib-0054] Larrick, J.W. , Yu, L. , Chen, J. , Jaiswal, S. and Wycoff, K. (1998) Production of antibodies in transgenic plants. Res. Immunol. 149, 603–608. 9835425 10.1016/s0923-2494(98)80013-8

[pbi12541-bib-0055] Larrick, J.W. , Yu, L. , Naftzger, C. , Jaiswal, S. and Wycoff, K. (2001) Production of secretory IgA antibodies in plants. Biomol. Eng. 18, 87–94. 11566600 10.1016/s1389-0344(01)00102-2

[pbi12541-bib-0056] Liu, Y. and Li, J. (2014) Endoplasmic reticulum‐mediated protein quality control in Arabidopsis. Front. Plant Sci. 5, 162. 24817869 10.3389/fpls.2014.00162 PMC4012192

[pbi12541-bib-0057] Loos, A. and Steinkellner, H. (2014) Plant glyco‐biotechnology on the way to synthetic biology. Front. Plant Sci. 5, 523. 25339965 10.3389/fpls.2014.00523 PMC4189330

[pbi12541-bib-0058] Lu, H. , Chandrasekar, B. , Oeljeklaus, J. , Misas‐Villamil, J.C. , Wang, Z. , Shindo, T. , Bogyo, M. *et al*. (2015) Subfamily‐specific fluorescent probes for cysteine proteases display dynamic protease activities during seed germination. Plant Physiol. 168, 1462–1475. 26048883 10.1104/pp.114.254466 PMC4528725

[pbi12541-bib-0059] Ma, J.K.‐C. (1988) Prevention of colonization of Strep mutans by local passive immunization with monoclonal antibodies in humans. J. Dent. Res. 67, 643–643.

[pbi12541-bib-0060] Ma, J.K.‐C. , Hiatt, A. , Hein, M. , Vine, N.D. , Wang, F. , Stabila, P. , van Dolleweerd, C. *et al*. (1995) Generation and assembly of secretory antibodies in plants. Science, 268, 716–719. 7732380 10.1126/science.7732380

[pbi12541-bib-0061] Ma, J.K.‐C. , Drake, P.M.W. and Christou, P. (2003) The production of recombinant pharmaceutical proteins in plants. Nat. Rev. Genet. 4, 794–805. 14526375 10.1038/nrg1177

[pbi12541-bib-0062] Ma, J.K.‐C. , Drossard, J. , Lewis, D. , Altmann, F. , Boyle, J. , Christou, P. , Cole, T. *et al*. (2015) Regulatory approval and a first‐in‐human phase I clinical trial of a monoclonal antibody produced in transgenic tobacco plants. Plant Biotechnol. J. 13, 1106–1120. 26147010 10.1111/pbi.12416

[pbi12541-bib-0063] Mason, H.S. , Ball, J.M. , Shi, J.J. , Jiang, X. , Estes, M.K. and Arntzen, C.J. (1996) Expression of Norwalk virus capsid protein in transgenic tobacco and potato and its oral immunogenicity in mice. Proc. Natl. Acad. Sci. USA, 93, 5335–5340. 8643575 10.1073/pnas.93.11.5335 PMC39246

[pbi12541-bib-0064] Mason, H.S. , Haq, T.A. , Clements, J.D. and Arntzen, C.J. (1998) Edible vaccine protects mice against *Escherichia coli* heat‐labile enterotoxin (LT): potatoes expressing a synthetic LT‐B gene. Vaccine. 16, 1336–1343. 9682399 10.1016/s0264-410x(98)80020-0

[pbi12541-bib-0065] McCormick, A.A. , Reddy, S. , Reinl, S.J. , Cameron, T.I. , Czerwinkski, D.K. , Vojdani, F. , Hanley, K.M. *et al*. (2008) Plant‐produced idiotype vaccines for the treatment of non‐Hodgkin's lymphoma: safety and immunogenicity in a phase I clinical study. Proc. Natl. Acad. Sci. USA, 105, 10131–10136. 18645180 10.1073/pnas.0803636105 PMC2481377

[pbi12541-bib-0066] Meuris, L. , Santens, F. , Elson, G. , Festjens, N. , Boone, M. , Dos Santos, A. , Devos, S. *et al*. (2014) GlycoDelete engineering of mammalian cells simplifies N‐glycosylation of recombinant proteins. Nat. Biotechnol. 32, 485–489. 24752077 10.1038/nbt.2885 PMC7039703

[pbi12541-bib-0067] Nochi, T. , Takagi, H. , Yuki, Y. , Yang, L. , Masumura, T. , Mejima, M. , Nakanishi, U. *et al*. (2007) Rice‐based mucosal vaccine as a global strategy for cold‐chain‐ and needle‐free vaccination. Proc. Natl. Acad. Sci. USA, 104, 10986–10991. 17573530 10.1073/pnas.0703766104 PMC1904174

[pbi12541-bib-0068] Nochi, T. , Yuki, Y. , Katakai, Y. , Shibata, H. , Tokuhara, D. , Mejima, M. , Kurokawa, S. *et al*. (2009) A rice‐based oral cholera vaccine induces macaque‐specific systemic neutralizing antibodies but does not influence pre‐existing intestinal immunity. J. Immunol. 183, 6538–6544. 19880451 10.4049/jimmunol.0901480

[pbi12541-bib-0069] Obembe, O.O. , Popoola, J.O. , Leelavathi, S. and Reddy, S.V. (2011) Advances in plant molecular farming. Biotechnol. Adv. 29, 210–222. 21115109 10.1016/j.biotechadv.2010.11.004

[pbi12541-bib-0070] Paul, M. , Reljic, R. , Klein, K. , Drake, P.M.W. , van Dolleweerd, C. , Pabst, M. , Windwarder, M. *et al*. (2014) Characterization of a plant‐produced recombinant human secretory IgA with broad neutralizing activity against HIV. MAbs, 6, 1585–1597. 25484063 10.4161/mabs.36336 PMC4622858

[pbi12541-bib-0071] Petruccelli, S. , Otegui, M.S. , Lareu, F. , Tran Dinh, O. , Fitchette, A.‐C. , Circosta, A. , Rumbo, M. *et al*. (2006) A KDEL‐tagged monoclonal antibody is efficiently retained in the endoplasmic reticulum in leaves, but is both partially secreted and sorted to protein storage vacuoles in seeds. Plant Biotechnol. J. 4, 511–527. 17309727 10.1111/j.1467-7652.2006.00200.x

[pbi12541-bib-0072] Piron, R. , Santens, F. , De Paepe, A. , Depicker, A. and Callewaert, N. (2015) Using GlycoDelete to produce proteins lacking plant‐specific N‐glycan modification in seeds. Nat. Biotechnol. 33, 1135–1137. 26544140 10.1038/nbt.3359

[pbi12541-bib-0073] Plomp, R. , Dekkers, G. , Rombouts, Y. , Visser, R. , Koeleman, C.A.M. , Kammeijer, G.S.M. , Jansen, B.C. *et al*. (2015) Hinge‐region O‐glycosylation of human immunoglobulin G3 (IgG3). Mol. Cell Proteomics, 14, 1373–1384. 25759508 10.1074/mcp.M114.047381 PMC4424406

[pbi12541-bib-0074] Qiu, X. , Wong, G. , Audet, J. , Bello, A. , Fernando, L. , Alimonti, J.B. , Fausther‐Bovendo, H. *et al*. (2014) Reversion of advanced Ebola virus disease in nonhuman primates with ZMapp. Nature, 514, 47–53. 25171469 10.1038/nature13777 PMC4214273

[pbi12541-bib-0075] Ramessar, K. , Rademacher, T. , Sack, M. , Stadlmann, J. , Platis, D. , Stiegler, G. , Labrou, N. *et al*. (2008) Cost‐effective production of a vaginal protein microbicide to prevent HIV transmission. Proc. Natl. Acad. Sci. USA, 105, 3727–3732. 18316741 10.1073/pnas.0708841104 PMC2268773

[pbi12541-bib-0076] Renukuntla, J. , Vadlapudi, A.D. , Patel, A. , Boddu, S.H. and Mitra, A.K. (2013) Approaches for enhancing oral bioavailability of peptides and proteins. Int. J. Pharm. 447, 75–93. 23428883 10.1016/j.ijpharm.2013.02.030 PMC3680128

[pbi12541-bib-0077] Robert, S. , Khalf, M. , Goulet, M.‐C. , D'Aoust, M.‐A. , Sainsbury, F. and Michaud, D. (2013) Protection of recombinant mammalian antibodies from development‐dependent proteolysis in leaves of *Nicotiana benthamiana* . PLoS ONE, 8, e70203. 23894618 10.1371/journal.pone.0070203 PMC3720903

[pbi12541-bib-0078] Sack, M. , Hofbauer, A. , Fischer, R. and Stoger, E. (2015a) The increasing value of plant‐made proteins. Curr. Opin. Biotechnol. 32, 163–170. 25578557 10.1016/j.copbio.2014.12.008 PMC6485345

[pbi12541-bib-0079] Sack, M. , Rademacher, T. , Spiegel, H. , Boes, A. , Hellwig, S. , Drossard, J. , Stoger, E. *et al*. (2015b) From gene to harvest: insights into upstream process development for the GMP production of a monoclonal antibody in transgenic tobacco plants. Plant Biotechnol. J. 13, 1094–1105. 26214282 10.1111/pbi.12438

[pbi12541-bib-0080] Sainsbury, F. , Lavoie, P.‐O. , D'Aoust, M.‐A. , Vézina, L.‐P. and Lomonossoff, G.P. (2008) Expression of multiple proteins using full‐length and deleted versions of cowpea mosaic virus RNA‐2. Plant Biotechnol. J. 6, 82–92. 17986176 10.1111/j.1467-7652.2007.00303.x

[pbi12541-bib-0081] Sarrion‐Perdigones, A. , Falconi, E.E. , Zandalinas, S.I. , Juarez, P. , Fernandez‐del‐Carmen, A. , Granell, A. and Orzaez, D. (2011a) GoldenBraid: an iterative cloning system for standardized assembly of reusable genetic modules. PLoS ONE, 6, e21622. 21750718 10.1371/journal.pone.0021622 PMC3131274

[pbi12541-bib-0082] Sarrion‐Perdigones, A. , Juarez, P. , Granell, A. and Orzaez, D . (2011b) Production of antibodies in plants. In: Antibody Expression and Production (Cell Engineering), Vol.7 ( Al‐Rubeai, M . ed), pp. 143–164. Dordrecht: Springer.

[pbi12541-bib-0083] Schnell, J. , Steele, M. , Bean, J. , Neuspiel, M. , Girard, C. , Dormann, N. , Pearson, C. *et al*. (2015) A comparative analysis of insertional effects in genetically engineered plants: considerations for pre‐market assessments. Transgenic Res. 24, 1–17. 25344849 10.1007/s11248-014-9843-7 PMC4274372

[pbi12541-bib-0084] Shaaltiel, Y. , Bartfeld, D. , Hashmueli, S. , Baum, G. , Brill‐Almon, E. , Galili, G. , Dym, O. *et al*. (2007) Production of glucocerebrosidase with terminal mannose glycans for enzyme replacement therapy of Gaucher's disease using a plant cell system. Plant Biotechnol. J. 5, 579–590. 17524049 10.1111/j.1467-7652.2007.00263.x

[pbi12541-bib-0085] Shaaltiel, Y. , Gingis‐Velitski, S. , Tzaban, S. , Fiks, N. , Tekoah, Y. and Aviezer, D. (2015) Plant‐based oral delivery of beta‐glucocerebrosidase as an enzyme replacement therapy for Gaucher's disease. Plant Biotechnol. J. 13, 1033–1040. 25828481 10.1111/pbi.12366

[pbi12541-bib-0086] Shil, P.K. , Kwon, K.C. , Zhu, P. , Verma, A. , Daniell, H. and Li, Q. (2014) Oral delivery of ACE2/Ang‐(1‐7) bioencapsulated in plant cells protects against experimental uveitis and autoimmune uveoretinitis. Mol. Ther. 22, 2069–2082. 25228068 10.1038/mt.2014.179 PMC4429699

[pbi12541-bib-0087] Steinkellner, H. and Castilho, A. (2015) *N*‐glyco‐engineering in plants: update on strategies and major achievements. Methods Mol. Biol. 1321, 195–212. 26082224 10.1007/978-1-4939-2760-9_14

[pbi12541-bib-0088] Strasser, R. , Castilho, A. , Stadlmann, J. , Kunert, R. , Quendler, H. , Gattinger, P. , Jez, J. *et al*. (2009) Improved virus neutralization by plant‐produced anti‐HIV antibodies with a homogeneous beta1,4‐galactosylated N‐glycan profile. J. Biol. Chem. 284, 20479–20485. 19478090 10.1074/jbc.M109.014126 PMC2742812

[pbi12541-bib-0089] Su, J. , Sherman, A. , Doerfler, P.A. , Byrne, B.J. , Herzog, R.W. and Daniell, H. (2015a) Oral delivery of Acid Alpha Glucosidase epitopes expressed in plant chloroplasts suppresses antibody formation in treatment of Pompe mice. Plant Biotechnol. J. 13, 1023–1032. 26053072 10.1111/pbi.12413 PMC4578979

[pbi12541-bib-0090] Su, J. , Zhu, L. , Sherman, A. , Wang, X. , Lin, S. , Kamesh, A. , Norikane, J.H. *et al*. (2015b) Low cost industrial production of coagulation factor IX bioencapsulated in lettuce cells for oral tolerance induction in hemophilia B. Biomaterials, 70, 84–93. 26302233 10.1016/j.biomaterials.2015.08.004 PMC4562874

[pbi12541-bib-0091] Tacket, C.O. (2007) Plant‐based vaccines against diarrheal diseases. Trans. Am. Clin. Climatol. Assoc. 118, 79–87. 18528491 PMC1863586

[pbi12541-bib-0092] Tacket, C.O. , Mason, H.S. , Losonsky, G. , Clements, J.D. , Levine, M.M. and Arntzen, C.J. (1998) Immunogenicity in humans of a recombinant bacterial antigen delivered in a transgenic potato. Nat. Med. 4, 607–609. 9585236 10.1038/nm0598-607

[pbi12541-bib-0093] Tacket, C.O. , Pasetti, M.F. , Edelman, R. , Howard, J.A. and Streatfield, S. (2004) Immunogenicity of recombinant LT‐B delivered orally to humans in transgenic corn. Vaccine. 22, 4385–4389. 15474732 10.1016/j.vaccine.2004.01.073

[pbi12541-bib-0094] Takaiwa, F. , Wakasa, Y. , Takagi, H. and Hiroi, T. (2015) Rice seed for delivery of vaccines to gut mucosal immune tissues. Plant Biotechnol. J. 13, 1041–1055. 26100952 10.1111/pbi.12423

[pbi12541-bib-0095] Takeyama, N. , Yuki, Y. , Tokuhara, D. , Oroku, K. , Mejima, M. , Kurokawa, S. , Kuroda, M. *et al*. (2015) Oral rice‐based vaccine induces passive and active immunity against enterotoxigenic *E. coli*‐mediated diarrhea in pigs. Vaccine. 33, 5204–5211. 26254309 10.1016/j.vaccine.2015.07.074

[pbi12541-bib-0096] Tokuhara, D. , Alvarez, B. , Mejima, M. , Hiroiwa, T. , Takahashi, Y. , Kurokawa, S. , Kuroda, M. *et al*. (2013) Rice‐based oral antibody fragment prophylaxis and therapy against rotavirus infection. J. Clin. Invest. 123, 3829–3838. 23925294 10.1172/JCI70266 PMC3754275

[pbi12541-bib-0097] Vézina, L.P. , Faye, L. , Lerouge, P. , D'Aoust, M.‐A. , Marquet‐Blouin, E. , Burel, C. , Lavoie, P.‐O. *et al*. (2009) Transient co‐expression for fast and high‐yield production of antibodies with human‐like N‐glycans in plants. Plant Biotechnol. J. 7, 442–455. 19422604 10.1111/j.1467-7652.2009.00414.x

[pbi12541-bib-0098] Virdi, V. , Coddens, A. , De Buck, S. , Millet, S. , Goddeeris, B.M. , Cox, E. , De Greve, H. *et al*. (2013) Orally fed seeds producing designer IgAs protect weaned piglets against enterotoxigenic *Escherichia coli* infection. Proc. Natl. Acad. Sci. USA, 110, 11809–11814. 23801763 10.1073/pnas.1301975110 PMC3718133

[pbi12541-bib-0099] Weintraub, J.A. , Hilton, J.F. , White, J.M. , Hoover, C.I. , Wycoff, K.L. , Yu, L. , Larrick, J.W. *et al*. (2005) Clinical trial of a plant‐derived antibody on recolonization of mutans streptococci. Caries Res. 39, 241–450. 15914988 10.1159/000084805

[pbi12541-bib-0100] Whaley, K.J. , Hiatt, A. and Zeitlin, L. (2011) Emerging antibody products and Nicotiana manufacturing. Hum. Vaccin. 7, 349–356. 21358287 10.4161/hv.7.3.14266 PMC3166493

[pbi12541-bib-0101] Wieland, W.H. , Orzaez, D. , Lammers, A. , Parmentier, H.K. and Schots, A. (2005) Display and selection of chicken IgA phage fragments. Vet. Immunol. Immunopathol. 110, 129–140. 16280167 10.1016/j.vetimm.2005.09.012

[pbi12541-bib-0102] Wurm, F.M. (2004) Production of recombinant protein therapeutics in cultivated mammalian cells. Nat. Biotechnol. 22, 1393–1398. 15529164 10.1038/nbt1026

[pbi12541-bib-0103] Wycoff, K.L. (2005) Secretory IgA antibodies from plants. Curr. Pharm. Des. 11, 2429–2437. 16026297 10.2174/1381612054367508

[pbi12541-bib-0104] Yoshiola, K.R.C.B. , Sato, K.R.C.B. , Gotanda, T.R.C.B. , Ito, A.N.A.T.I.O.F.A.I.N.D.S.C.I.A.N.D.T. , Isogai, E.S.D. , Takehara, K. and Maehara, N . (2012) Oral composition containing Interferon‐alpha. Google Patents.

[pbi12541-bib-0105] Zeitlin, L. , Olmsted, S.S. , Moench, T.R. , Co, M.S. , Martinell, B.J. , Paradkar, V.M. , Russell, D.R. *et al*. (1998) A humanized monoclonal antibody produced in transgenic plants for immunoprotection of the vagina against genital herpes. Nat. Biotechnol. 16, 1361–1364. 9853620 10.1038/4344

